# National digital pathology projects in Switzerland: A 2023 update

**DOI:** 10.1007/s00292-023-01259-5

**Published:** 2023-11-21

**Authors:** Rainer Grobholz, Andrew Janowczyk, Ana Leni Frei, Mario Kreutzfeldt, Viktor H. Koelzer, Inti Zlobec

**Affiliations:** 1 Medical Faculty, University of Zurich, Zurich, Switzerland; 2 Institute of Pathology, Kantonsspital Aarau, Tellstr. 25, 5001 Aarau, Switzerland; 3 Department of Biomedical Engineering, Emory University, Atlanta, GA USA; 4 Department of Oncology, Division of Precision Oncology, University Hospital of Geneva, Geneva, Switzerland; 5 Department of Diagnostics, Division of Clinical Pathology, University Hospital of Geneva, Geneva, Switzerland; 6 Institute for Tissue Medicine and Pathology, University Bern, Bern, Switzerland; 7 Department of Pathology and Immunology, Division of Clinical Pathology, University & University Hospitals of Geneva, Geneva, Switzerland; 8 Department of Pathology und Molecular Pathology, University Hospital Zurich, University of Zurich, Zurich, Switzerland

**Keywords:** Digital pathology, National strategies, Digital image analysis, National Guidelines, National Surveys, Digitale Pathologie, Nationale Strategien, Digitale Bildanalyse, Nationale Leitlinien, Nationale Umfragen

## Abstract

The Swiss Digital Pathology Consortium (SDiPath) was founded in 2018 as a working group of the Swiss Society for Pathology with the aim of networking, training, and promoting digital pathology (DP) at a national level. Since then, two national surveys have been carried out on the level of knowledge, dissemination, use, and needs in DP, which have resulted in clear fields of action. In addition to organizing symposia and workshops, national guidelines were drawn up and an initiative for a national DP platform actively codesigned. With the growing use of digital image processing and artificial intelligence tools, continuous monitoring, evaluation, and exchange of experiences will be pursued, along with best practices.

## Abstract (de)

Das Schweizer Konsortium für Digitale Pathologie (Swiss Digital Pathology Consortium, SDiPath) wurde 2018 als Arbeitsgruppe der Schweizerischen Gesellschaft für Pathologie gegründet mit der Zielsetzung der Vernetzung, Fortbildung und Förderung der digitalen Pathologie (DP) auf nationaler Ebene. Seither wurden 2 nationale Umfragen zum Kenntnisstand, der Verbreitung, Verwendung und der Bedürfnisse in der DP durchgeführt, aus welchen sich klare Handlungsfelder ergeben haben. Neben der Ausrichtung von Symposien und Workshops wurden nationale Leitlinien erarbeitet und eine Initiative für eine nationale DP-Plattform aktiv mitgestaltet. Mit dem zunehmenden Einsatz von digitaler Bildverarbeitung und künstlicher Intelligenz werden die kontinuierliche Begleitung, die Evaluation und der Austausch von Erfahrungen sowie bewährten praktischen Ansätzen weiterverfolgt.

Digitization in medicine is advancing in leaps and bounds, particularly in the areas of data processing, data transmission, and accessibility of stored data. In the field of diagnostics, computer-aided systems have been in operation for many years in the context of research or, in some cases, clinical applications. Systems first developed in the early 1980s used video technology to enable live transmission of image material for collaborative diagnostics between hospitals. During this time, the term “telepathology” was coined under the generic term “telemedicine” [1]. As early as 1986, “robotic dynamic telepathology” was demonstrated for the first time, using satellite-based remote control of a motorized microscope and simultaneous video image transmission [1]. From these first steps, digitization in pathology slowly evolved together with the new possibilities offered by computer technology. However, the term “digital pathology” (DP) was only first coined in 1999 [2]. Interestingly, since both terms were coined, the PubMed database has listed only 1346 (1986–07/2023) articles for the search term “telepathology,” while the term “digital pathology” has produced 2583 (1999–07/2023) articles, suggesting a strong recent uptake by the community (Fig. 1). The first vision of scanned slide preparations and an “electronic microscope” for use in consultation pathology was expressed in the literature in the year 2000 [3]. Due to the advancement of technology and the consistent development of the digitization of slides through scanner technology, the systems were able to reach market maturity [4]. Approval for diagnostic operation of the scanners was first granted in Europe through CE certification in 2014, and in the USA by the Food and Drug Administration in 2017 [5]. This paved the way for their entry into daily clinical practice.

**Fig. 1:**
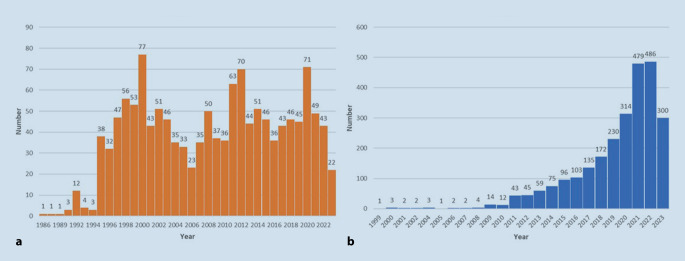
**a** PubMed-listed publications for “telepathology” 1986–07/2023 (*n* = 1346), **b** PubMed-listed publications for “digital pathology” 1999–07/2023 (*n* = 2583)

Due to the increasing adoption of DP in Switzerland, the “Swiss Digital Pathology Consortium” (SDiPath) was founded in 2018 as a working group of the Swiss Society of Pathology (SSPath), and currently has over 170 members. The objectives of SDiPath are:

- networking and exchange of all professional groups involved in DP (pathologists, researchers, laboratory staff, IT technologists, developers);
- continuing education in the special aspects of DP;
- platform and coordination of research projects; and
- promotion of digitization in pathology on a national level.

In addition to its own website (www.sdipath.ch), information about latest articles, activities, and training is shared in a quick and easy manner via a Google Groups mailing list.

## National surveys on digital pathology

At the beginning of SDiPath’s work, a survey was conducted in 2019 to determine the current state of knowledge and dissemination of DP in Switzerland. For this purpose, a questionnaire was generated with 12 questions focusing on a) personal experiences, b) experiences with digital sections and image analysis systems, c) opinions on the development of DP, and d) general advantages and disadvantages of DP [6]. During an SSPath slide seminar with the topic “immunohistochemistry,” the questionnaire was distributed and completed by 134 of 159 participants.

This survey showed that in international comparison, there was a relatively high level of experience in Switzerland in the handling and usage of digital sectioning preparations (89% of respondents), and limited experience in the use of image analysis systems (34% of respondents). Attitudes toward DP were positive (82%), and 66% could envision performing diagnostics completely digitally. Only 10% showed a completely negative attitude. Advantages of DP mentioned were availability of slides, reproducibility of measurements, remote access to sections, obtaining second opinions, image analysis, and training and education. Disadvantages mentioned were the IT infrastructure, the insufficient possibility of digitizing special methods such as polarization, the costs/billing, the different image formats, the non-existing standardization, along with the loss of knowledge in the use of microscopes.

From these results, the need for knowledge transfer, training, and visibility of DP through SDiPath could be clearly seen.

In 2018–2023, a total of seven symposia, three workshops, and one slide seminar on digital pathology were hosted. SDiPath also participated in hosting one DP course and two symposia on DP in the three-country Germany–Austria–Switzerland (DACH) region.

The symposia were focused on exchange of experiences, scientific projects, and keynote lectures. Two events were held together with industry, providing attendees with an overview of the current commercial DP platforms/scanners and image processing systems as well as artificial intelligence solutions.

With the COVID-19 pandemic, work patterns in pathology changed abruptly and digitization has received a significant boost in times of recommended and/or mandated home office work. To understand the impact of the pandemic on the status of DP, a renewed national survey on this topic was conducted [7]. For this purpose, a questionnaire with 21 questions on a) general working environment and experiences with DP; b) DP system design, structure, and use; c) DP system application during the pandemic; and d) specific staffing, technical, and organizational challenges was used.

**Fig. 2:**
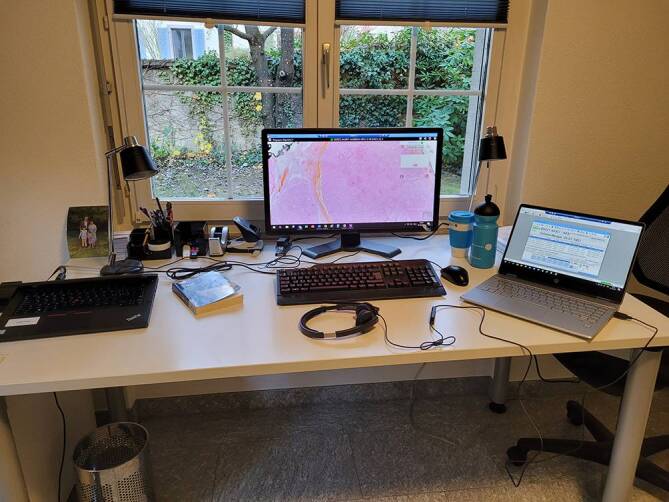
Home office workplace for remote diagnostics by digital pathology (© Dr. Alejandra Magagna, with kind permission)

Due to the pandemic situation, the questionnaire was created electronically in Google Forms, and all members of SDiPath and SSPath were invited via email. Non-members of the societies were invited via their respective supervisors. A total of 74 participants (18.6% of SSPath members) completed the questionnaire, and 40.5% reported experience with > 100 digital slices in routine diagnostics, with 39.2% having a DP system established and in use. The proportion of users for primary diagnostics was 13.5% before and 30% during the pandemic. Notably, DP offered the option of home office (Fig. 2), which was used by 40.6% of participants. Challenges included obtaining additional hardware (monitors, additional scanners) and establishing the remote connection. Due to the unanticipated pandemic, only 20.5% had a standard operating procedure in place for routine DP use.

## National Guidelines for DP

**Fig. 3:**
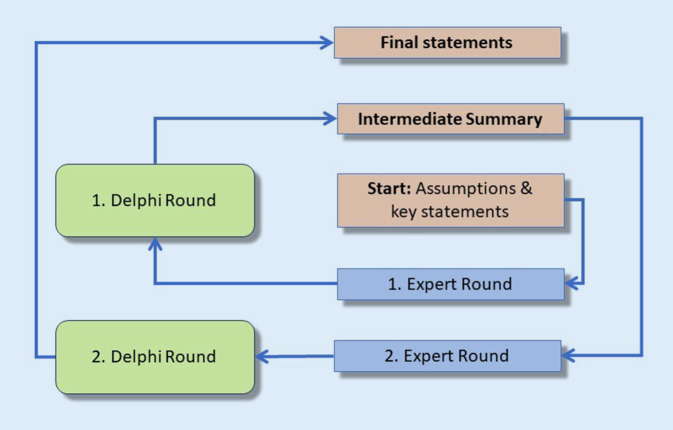
Development of the guidelines with the help of the Delphi method

From these results, it was evident that compared to the first survey, digitization and use in routine diagnostics had continued to increase. Due to the lack of national guidelines, SDiPath convened four working groups to develop current guidelines based on previously existing guidelines from other national societies of pathology [8–14]. The evaluation was performed using a Delphi method, in which the participating experts vote on the respective points. In case of discordance, the individual points are discussed, revised, and put to another round of voting. This process is repeated until a consensus is reached for all points (Fig. 3).

In addition to adapting the guidelines to national circumstances, recommendations for dealing with digital image analysis (DIA) and artificial intelligence (AI) tools were developed in a separate chapter (publication under review, preprint available: https://www.medrxiv.org/content/10.1101/2023.09.15.23295616v1). These recommendations are also elaborated in the article by S. Berezowska in this volume of *Die Pathologie*.

## National study on determination of the tumor cell fraction

Since it was apparent from the previous surveys that there is a great desire for support and standardization of quantification in pathology, a national study was conducted to estimate the tumor cell fraction with and without the use of a DIA tool. For this purpose, one representative hematoxylin/eosin-stained image of each of 10 colorectal tumors was selected at a 400 × magnification, each with a different tumor cell content. Tumor cell quantification was performed in two stages: a) manually and b) aided by DIA. As part of an SSPath slide seminar, 69 participants took part in the study. In the first stage, participants were asked to indicate the tumor cell fraction without DIA support in the 10 tumor images. In the second stage, the same images were shown again, but this time with the result of the DIA algorithm overlaid. Finally, for each case, participants indicated whether DIA was helpful in the assessment. The results showed that interobserver variability significantly decreased and participants were significantly more comfortable with their estimation result using DIA than without [15].

## National Digital Pathology Initiative

The increasing digitization offers the opportunity at this stage to establish unified national platforms to enable regulated data exchange. This is especially helpful for research and daily clinical work, as data are available in a standardized form. Under the leadership of V. H. Koelzer, the five Swiss medical faculties and SDiPath have worked out the establishment of a Swiss Digital Pathology Infrastructure (SDPI), which, once established, will provide in a first step the generation of uniform slide scans and a unified platform for anonymized digital section preparations and clinical data for a common data exchange [16]. In a second step, expansion to non-university facilities is planned. The SDPI has been included in the roadmap of research infrastructures of the State Secretariat for Education, Research, and Innovation with a funding volume of CHF 18.4 million (https://www.sbfi.admin.ch/sbfi/de/home/dienstleistungen/publikationen/publikationsdatenbank/roadmap_forschungsinfrastrukturen_2023.html). The final funding decision is expected in February 2024.

## Outlook

SDiPath will continue to accompany the further development of DP and will promote and support digitization by regular symposia and workshops. With the increasing use of DIA and AI tools in routine diagnostics, the question of invoicing of this service moves into the foreground, which requires the development of appropriate recommendations. The digitization in pathology will continue to grow and active support is therefore of great importance.

## Declarations

For this article no studies with human participants or animals were performed by any of the authors. All studies mentioned were in accordance with the ethical standards indicated in each case.

The supplement containing this article is not sponsored by industry.
